# The Emerging Role of microRNAs in Aquaporin Regulation

**DOI:** 10.3389/fchem.2018.00238

**Published:** 2018-06-21

**Authors:** André Gomes, Inês V. da Silva, Cecília M. P. Rodrigues, Rui E. Castro, Graça Soveral

**Affiliations:** ^1^Research Institute for Medicines (iMed.ULisboa), Faculty of Pharmacy, Universidade de Lisboa, Lisbon, Portugal; ^2^Department Bioquimica e Biologia Humana, Faculty of Pharmacy, Universidade de Lisboa, Lisbon, Portugal

**Keywords:** aquaporin, miRNA, gene expression regulation, post-transcriptional modulation, membrane proteins, permeability, disease

## Abstract

Aquaporins (AQPs) are membrane channels widely distributed in human tissues. AQPs are essential for water and energy homeostasis being involved in a broad range of pathophysiological processes such as edema, brain injury, glaucoma, nephrogenic diabetes insipidus, salivary and lacrimal gland dysfunction, cancer, obesity and related metabolic complications. Compelling evidence indicates that AQPs are targets for therapeutic intervention with potential broad application. Nevertheless, efficient AQP modulators have been difficult to find due to either lack of selectivity and stability, or associated toxicity that hamper *in vivo* studies. MicroRNAs (miRNAs) are naturally occurring small non-coding RNAs that regulate post-transcriptional gene expression and are involved in several diseases. Recent identification of miRNAs as endogenous modulators of AQP expression provides an alternative approach to target these proteins and opens new perspectives for therapeutic applications. This mini-review compiles the current knowledge of miRNA interaction with AQPs highlighting miRNA potential for regulation of AQP-based disorders.

## Introduction

Aquaporins (AQPs) are membrane channels that facilitate diffusion of water and small molecules (e.g., glycerol) through cell membranes driven by osmotic or solute gradients. The 13 isoforms (AQP0-12) expressed in mammals are crucial for water homeostasis and energy balance, which in turn influence survival and adaptation of living organisms. AQPs participate in many physiological processes such as renal water absorption, brain water homeostasis, skin hydration, intestinal permeability, cell proliferation, migration and angiogenesis, and oxidative stress response (Verkman, 2012; Pelagalli et al., 2016; Rodrigues et al., 2016). This suggests that their role may go far beyond the simple facilitation of membrane permeability. Indeed, over the years the importance of AQPs in health and disease has gained the attention of several research groups around the world; there is now compelling evidence that aquaporins are drug targets with potential broad application (Soveral et al., 2016). Modulators of AQPs expression or function with high selectivity and low side-toxicity are anticipated to have high value for the treatment of AQP-related disorders such as edema, brain injury, glaucoma, nephrogenic diabetes insipidus, salivary and lacrimal gland dysfunction, cancer and obesity, among others (Verkman et al., 2014; Soveral et al., 2016).

Although several potential AQP modulators have been reported and patented for use in diagnostic and therapeutics (Beitz et al., 2015; Soveral and Casini, 2017), their lack of selectivity and toxic side effects has hampered application in clinical trials. In addition, the protein structural conformation with channel pore access restrictions renders the molecule difficult to target and has slowed the progress of AQP drug discovery (Verkman et al., 2014; Madeira et al., 2016).

The recent recognition of AQP targeting by microRNAs (miRNAs) has opened new avenues for drug development. Here, we summarize updated information on the role of miRNAs in AQP-selective regulation and discuss their usefulness to tailor specific AQP-based therapeutics.

## Overview of miRNA biogenesis and function

**Figure 1:**
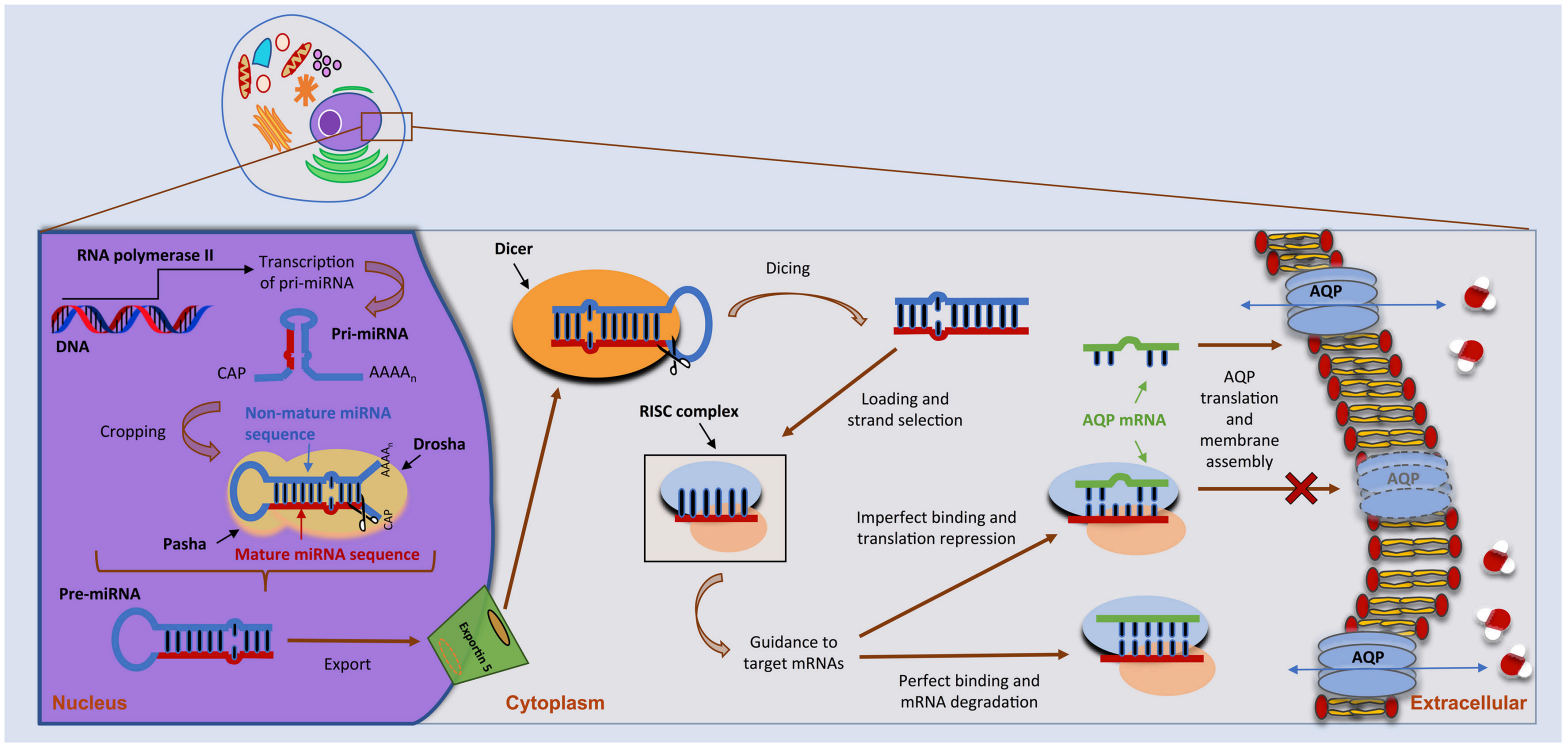
miRNA biogenesis and mode of action. miRNA biogenesis embodies a multistep process catalyzed by specific RNA polymerases. miRNAs are initially transcribed as a long, capped and polyadenylated pri-miRNA, cropped by the Drosha complex into a hairpin pre-miRNA. Following translocation to the cytoplasm by Exportin-5, the pre-miRNA is further processed by the Dicer complex, generating a ~22-nucleotide mature miRNA–miRNA duplex. The guide strand is then selected by the Argonaute protein and integrated into an RNA-induced silencing complex (RISC) to form the miRNA–RISC. This will act on target mRNAs, including aquaporin (AQP) mRNAs, by binding to the 3′-UTR and leading to translational inhibition or mRNA degradation (see text for more details).

miRNAs are small, single-stranded non-coding RNAs with important functions in the post-transcriptional control of gene expression (Ha and Kim, 2014; Christopher et al., 2016; Vishnoi and Rani, 2017). In humans, miRNA biogenesis follows a multi-step process depicted in Figure 1. miRNAs are firstly transcribed in the nucleus by RNA polymerase II (Pol II) as long primary transcripts (pri-miRNAs), exhibiting a double-stranded hairpin loop structure (Ha and Kim, 2014). This stem loop is then cropped by nuclear RNase III Drosha to release a small hairpin-shaped RNA of ~65 nucleotides in length (pre-miRNA). Next, the pre-miRNA is exported to the cytoplasm through a nuclear pore complex comprising protein exportin 5 and further processed by RNase III endonuclease DICER near the terminal loop, liberating a small ~22 nucleotides in length RNA duplex. This duplex is then loaded into the miRNA-induced silencing complex (miRISC), unwounded, and the mature miRNA transferred to Argonaute (AGO) proteins within the complex. Following its assembly in the miRISC, the miRNA will target one or multiple mRNAs, leading to translational repression or, in particular cases, to mRNA degradation (Pereira et al., 2013; Ha and Kim, 2014; Vishnoi and Rani, 2017). Of note, miRNAs may also act as transcriptional or splicing regulators, within the nucleus (Hwang et al., 2007), and be involved in genetic exchange with adjacent cells, through exosomes (Valadi et al., 2007). Approximately 60% of protein-coding genes are influenced by miRNAs (Friedman et al., 2009) that play crucial roles in several biological processes, including control of cell cycle and differentiation, proliferation and metabolism. As such, miRNA deregulation is being increasingly associated with several human pathologies.

miRNAs might embody prospective therapeutic targets. We have recently shown that miR-21 is systematically increased in animal models and in human patients with steatohepatitis, thus contributing for disease pathogenesis. In contrast, miR-21 abrogation significantly improved steatosis, inflammation and fibrosis, as well as overall lipid and cholesterol metabolism (Rodrigues et al., 2017). Other studies have similarly shown that miRNA functional manipulation *in vivo* can impact on metabolic phenotypes and even reverse the course of insulin resistance and diabetes (Sethupathy, 2016). These results suggest that miRNA-based therapies may become a viable strategy for treating a broad range of disorders such as cancer and cardiovascular disease, among others (van Rooij and Kauppinen, 2014; Adams et al., 2017). Further, in oncology the aim is to downregulate or block the function of oncogenic miRNAs and/or upregulate expression of tumor suppressor miRNAs, for which different miRNA-targeting strategies have been proposed (as reviewed in Ling et al., 2013; Li and Rana, 2014; Robb et al., 2017). Replacement of tumor suppressor miRNAs typically involves the introduction of synthetic miRNA mimics or miRNA expression vectors. In this regard, a synthetic miRNA mimic based on the sequence of the miR-15/16 family is being evaluated in a clinical trial to treat patients with malignant pleural mesothelioma and advanced non-small cell lung cancer (van Zandwijk et al., 2017). As for inhibition of oncogenic miRNAs overexpressed in cancer, the top approaches being investigated include expression vectors (miRNA sponges), small-molecule inhibitors and antisense oligonucleotides (ASOs or antagomiRs) (Robb et al., 2017). Miravirsen (Santaris Pharma A/S) is a typical example of the later, inhibiting miR-122 function in the liver that is essential for the replication of the hepatitis C virus (HCV). A Phase II clinical trial showed that miravirsen is able to reduce HCV RNA levels in patients (Janssen et al., 2013).

In parallel with therapeutic targeting, circulating miRNA patterns are associated with metabolic, neurodegenerative and infectious pathologies (Keller et al., 2015; Mirra et al., 2015, 2018; Verma et al., 2016), making miRNAs attractive disease biomarkers and allowing the prospective implementation of personalized therapies (Mirra et al., 2018). Nonetheless, the use of miRNAs as either therapeutic targets or disease biomarkers still requires extensive optimization and validation.

## Aquaporin targeting by miRNAS

The discovery of miRNAs as endogenous modulators of AQPs offers a potential therapeutic approach for the regulation AQP-related disorders. Below, we address the current knowledge of miRNA interaction with AQP isoforms and the potential advantage for AQP-related pathologies (Table 1).

**Table 1:** Interaction of different miRNAs with AQPs in several pathophysiological conditions.

**Gene**	**miRNA**	**Tissue**	**Disease/condition**	**References**
AQP1	29a	Colon	IBS	Chao et al., 2017
	126-5p	Lung	Acute lung injury	Tang et al., 2016
	144-3p	Lung	Acute lung injury	Li et al., 2018
	320a	Brain	Cerebral ischemia	Sepramaniam et al., 2010
		Spinal cord	Spinal cord edema	Li et al., 2016
	320	Breast	Breast cancer	Luo et al., 2018
	666	Liver	Cirrhosis	Huebert et al., 2011
	708	Liver	Cirrhosis	Huebert et al., 2011
AQP2	32	Kidney	Water reabsorption	Kim et al., 2015
	137	Kidney	Water reabsorption	Kim et al., 2015; Ranieri et al., 2018
AQP3	1	Epidermis	Wound healing	Banerjee and Sen, 2015
	29a	Colon	IBS	Chao et al., 2017
	124	Liver	HCC	Chen et al., 2018
	185-5p	Epidermis	SCC	Ratovitski, 2013
	874	Stomach	GC	Jiang et al., 2014
		Intestine	Intestinal ischemic injury	Zhi et al., 2014
		Pancreas	PDAC	Huang et al., 2017
AQP4	19a	Brain	Astrocyte connectivity	Jullienne et al., 2018
	29b	Brain	Cerebral ischemia	Wang et al., 2015
	130a	Brain	Cerebral ischemia	Sepramaniam et al., 2012
		Brain	AD	Zhang et al., 2017
	130b	Brain	Cerebral ischemia	Zheng et al., 2017b
	145	Brain	Cerebral ischemia	Zheng et al., 2017a
	203	Lung	Asthma	Jardim et al., 2012
	224	Brain	Astrocyte connectivity	Jullienne et al., 2018
	320a	Brain	Cerebral ischemia	Sepramaniam et al., 2010
		Brain	Epilepsy	Song et al., 2015
		Brain	Glioma	Xiong et al., 2018
AQP5	21	Gallbladder	Gallbladder carcinoma	Sekine et al., 2013
	96	Lung	Sepsis	Zhang et al., 2014; Rump and Adamzik, 2018
	330	Lung	Sepsis	Zhang et al., 2014; Rump and Adamzik, 2018
AQP8	16	Colon	Ulcerative colitis	Min et al., 2013
	29a	Colon	IBS	Chao et al., 2017
	195	Colon	Ulcerative colitis	Min et al., 2013
	330	Colon	Ulcerative colitis	Min et al., 2013
	424	Colon	Ulcerative colitis	Min et al., 2013
	612	Colon	Ulcerative colitis	Min et al., 2013
AQP9	22	Liver	Diabetes	Karolina et al., 2014
	23a	Liver	Diabetes	Karolina et al., 2014

*AD, Alzheimer's disease; HCC, hepatocellular carcinoma; IBS, irritable bowel syndrome; GC, gastric cancer; PDAC, pancreatic ductal adenocarcinoma; SCC, squamous cell carcinoma*.

AQPs are specialized water and/or glycerol channels expressed in various tissues including the kidney, lung, gastrointestinal tract, brain, adipose tissue and liver (Verkman, 2012) and are implicated in water imbalance disorders, such as edema.

AQP1 and AQP4 are associated with cerebral edema (Griesdale and Honey, 2004; Zador et al., 2007), and their modulation may improve the outcome of cerebral disorders such as cytotoxic and vasogenic edema, stroke and traumatic brain injury (Papadopoulos and Verkman, 2007; Zador et al., 2007). Interestingly, miRNA deregulation has also been reported in cerebral ischemia (Koutsis et al., 2013; Ouyang et al., 2013; Di et al., 2014), a condition that can induce cerebral edema (Marmarou, 2007). miR-320a was reported to inhibit *AQP1* and *AQP4* gene expression both *in vitro* and *in vivo* in a cerebral ischemia rat model (Sepramaniam et al., 2010), whereas anti-miR-320a upregulated *AQP1* and *AQP4* expression with consequent reduction of infarct volume (Sepramaniam et al., 2010). The inhibitory effect of miR-320a on *AQP4* expression was also confirmed in astrocyte primary cultures from brain tissue of epileptic rats (Song et al., 2015), a condition that may induce cytotoxic cerebral edema. In addition, in a rat model of spinal cord edema, downregulation of *AQP1* at the blood–spinal cord barrier by miR-320a showed to positively affect spinal cord edema after ischemia reperfusion injury (Li et al., 2016). These findings suggest that miR-320a can be used as modulator of AQP1 and AQP4 in cerebral and spinal cord edema.

Further studies identified miR-130a as a transcriptional repressor of *AQP4* M1 isoform in human astrocytes (Sepramaniam et al., 2012). This transcript shows higher expression and function in the human brain under ischemic conditions compared to *AQP4* M23 (Hirt et al., 2009). Modulation of miR-130a and subsequent influence on *AQP4* M1 gene and protein expression may be used to reduce cerebral infarct and promote ischemic recovery (Sepramaniam et al., 2012). Additionally, *AQP4* down-regulation by miR-145 (Zheng et al., 2017a), miR-130b (Zheng et al., 2017b) and miR-29b (Wang et al., 2015) revealed the protecting role of these miRNAs against ischemic stroke. A recent study demonstrated that *AQP4* silencing in rat astrocyte primary cultures was associated with an increase of miR-224 and miR-19a expression, and this could be a molecular mechanism responsible for decreased astrocyte connectivity and water mobility in the brain (Jullienne et al., 2018).

AQP1 is also expressed in the lung alveolar epithelia and plays an important role in lung fluid transport and alveolar fluid clearance (King et al., 1996). Increased alveolar capillary membrane permeability, apoptosis of alveolar epithelial cells, inflammation and edema are characteristics of acute lung injury. In a mouse model of lipopolysaccharide-induced acute lung injury, miR-126-5p was down-regulated while AQP1 and epithelial sodium channel (ENaC) protein expression was reduced in alveolar type II cells (Tang et al., 2016). AQP1 and ENaC reduction was attenuated when miR-126-5p was overexpressed, suggesting that miR-126-5p may ameliorate dysfunction of alveolar fluid clearance by maintaining the activity of both AQP1 and ENaC. An opposite effect was promoted by miR-144-3p in acute lung injury mice and in a lung epithelial carcinoma cell line, where AQP1 mRNA and protein expression were both decreased when miR-144-3p was overexpressed, reducing lung epithelial cell apoptosis (Li et al., 2018).

AQP1 plays an important role in cell migration, angiogenesis, wound healing and tumor growth (Saadoun et al., 2002a; Tomita et al., 2017). It is highly expressed in cancer tissues and often associated with worse prognosis (Papadopoulos and Saadoun, 2015). miR-320 was shown to negatively regulate AQP1 expression and to reduce cell proliferation, migration, and invasion of breast cancer cells (Luo et al., 2018). The role of AQP1 in angiogenesis, fibrosis and portal hypertension in cirrhotic mice has been investigated in AQP1 knockout mice, which showed reduced angiogenesis and fibrosis. The osmotically sensitive miR-666 and miR-708 are decreased in cirrhosis and were found to regulate AQP1 expression, suggesting its modulation as a therapeutic strategy in chronic liver disease (Huebert et al., 2011).

AQP2 is expressed in kidney collecting duct epithelial cells where the high transepithelial water permeability accounts for fluid retention and urine concentration. Water reabsorption via AQP2 is controlled by vasopressin, which triggers AQP2 trafficking to the apical plasma membrane (short-term regulation) or increases transcription of *AQP2* gene (long-term regulation) (Nielsen et al., 2000). Two AQP2-targeting miRNAs, miR-32 and miR-137, were reported to decrease *AQP2* expression in kidney collecting duct cells independently of vasopressin regulation (Kim et al., 2015). *AQP2* targeting by miR-137 has recently been correlated with impaired response to vasopressin and reduction of urine concentration via the calcium-sensing receptor (CaSR). Once activated by high external calcium, CaSR promotes the synthesis of miRNA-137 and increases AQP2 ubiquitination and proteasomal degradation resulting in reduced AQP2 mRNA translation (Ranieri et al., 2018).

AQP3 is expressed in epidermal keratinocytes acting as a skin-hydration protein due to its ability to increase glycerol cellular content (Hara and Verkman, 2003). However, AQP3 is aberrantly expressed in different tumors (Papadopoulos and Saadoun, 2015) and its suppression has been proposed as a potential tool to reduce epidermal cell migration, proliferation and tumorigenicity (Hara-Chikuma and Verkman, 2008). AQP3-targeting by miRNAs resulted in decreased cell differentiation in different cancers, such as in squamous cell carcinoma by miR-185-5p (Ratovitski, 2013), gastric adenocarcinoma (Jiang et al., 2014) and pancreatic ductal adenocarcinoma (Huang et al., 2017) by miR-874, and hepatocellular carcinoma by miR-124 (Chen et al., 2018). In addition, miR-1 was proposed to indirectly target AQP3 impairing keratinocyte migration (Banerjee and Sen, 2015).

AQP3 has also an established role in transepithelial water transport in the colon, along with AQP1 and AQP8 (Laforenza, 2012; Zhao et al., 2016). Altered water secretion or absorption in the colon is linked to gut disorders such as irritable bowel syndrome (IBS), where increased intestinal permeability due to disruption of intestinal tight junctions contributes to diarrhea and abdominal pain. It has been reported that *AQP3* silencing leads to impairment of intestinal barrier integrity possibly by increasing paracellular permeability via an opening of the tight junction complex (Zhang et al., 2011) where miR-874 is involved through AQP3 targeting (Zhi et al., 2014; Su et al., 2016). Analysis of intestinal tissue samples from patients with IBS revealed that miR-29 reduces the expression of critical signaling molecules involved in the regulation of intestinal permeability (Zhou et al., 2015). The finding that AQP1, AQP3 and AQP8 are down-regulated by miR-29a in rat colon tissues, and increased by anti-miR-29a (Chao et al., 2017) unveils a potential tool to restore intestinal permeability via miR-29 blockage and AQP up-regulation.

AQP4 is mainly expressed in the brain with a polarized distribution in the perivascular endfeet of astrocytes. There is strong evidence that AQP4 mislocalization contributes to the excessive accumulation of amyloid-β in brain found in Alzheimer's disease (AD) (Yang et al., 2012). In a recent study, miR-130a restored AQP4 polarity by repressing the transcriptional activity of *AQP4* M1 decreasing the *AQP4* M1/M23 ratio (Zhang et al., 2017), thus protecting against AD. In addition to normal astrocytes, AQP4 is also expressed in human astrocytomas where the level of expression correlates with tumor aggressiveness (Saadoun et al., 2002b; Verkman et al., 2014). In glioma cells, miR-320a overexpression down-regulates AQP4 and diminishes cell invasion and migration, suggesting it could be used as a therapeutic target to suppress the aggressive capacity of this tumor (Xiong et al., 2018). Interestingly, AQP4 was found to be up-regulated in bronchial epithelial cells from asthmatic donors, following down-regulation of miR-203, together with pro-inflammatory genes (Jardim et al., 2012). The role of AQP4 in asthma is not clear, but since the progression of asthma usually includes edema, a contribution to fluid clearance cannot be ruled out.

AQP5 is a selective water channel important for saliva production and airway fluid clearance (Song and Verkman, 2001; Delporte et al., 2016). In the lung of rats after LPS-induced sepsis, decreased AQP5 gene and protein expression correlates with up-regulation of miR-96 and miR-330 and establishment of pulmonary edema (Zhang et al., 2014). AQP5 is also involved in cell proliferation, migration and invasion (Papadopoulos and Saadoun, 2015; Direito et al., 2016). AQP5 up-regulation in different cancer tissues together with markers of cancer progression suggests its involvement in cancer signaling pathways and highlights its potential as promising target for cancer therapy (Direito et al., 2016, 2017). *AQP5* expression in gallbladder carcinoma was regulated by miR-21 and correlated with early-stage tumor progression with favorable prognosis (Sekine et al., 2013), suggesting novel potential drug targets for this malignancy.

AQP8 is expressed in the epithelial cells of the intestine (Laforenza, 2012). In colon samples of ulcerative colitis patients, AQP8 mRNA and protein were found three-fold decreased. A search for candidate target miRNAs revealed miR-16, miR-195, miR-424, miR-612, and miR-330 as putative down-regulators of AQP8 expression (Min et al., 2013).

AQP7 and AQP9 transport glycerol in addition to water (aquaglyceroporins) and are involved in fat metabolism in the adipose and liver tissues (Hibuse et al., 2006; Madeira et al., 2015). In fasting conditions, when triglyceride lipolysis occurs, AQP7 facilitates glycerol efflux from adipose tissue into the circulation, which is taken up in the liver via AQP9 to be used for gluconeogenesis (Rodriguez et al., 2011). AQP7 and AQP9 coordinated function is crucial for energy homeostasis and deregulation has been implicated in obesity and diabetes (Rodriguez et al., 2014; da Silva and Soveral, 2017). Selective modulation of AQP7 and AQP9 may constitute a promising approach for controlling obesity and metabolic-related disorders (da Silva et al., 2018). Among the candidate miRNA regulators of adipogenesis and gluconeogenesis, miR-22 and miR-23a showed to reduce *AQP9* expression in liver cells, suggesting a potential application for glycaemia control in diabetic patients (Karolina et al., 2014).

## Final remarks

The wide distribution of the various AQP-isoforms in mammalian tissues and their implication in a broad range of pathophysiological conditions makes AQPs exciting drug targets for novel therapies. Yet, with the exception of a few small molecules, no modulators of AQPs are available for *in vivo* use (Soveral and Casini, 2017). The recent discovery of miRNAs as endogenous regulators of AQP expression highlights an alternative and indirect approach to selectively target AQPs through modulation of signal transduction pathways. Moreover, since miRNA-targeting oligonucleotides can be chemically modified to enhance their pharmacokinetic/pharmacodynamic properties, targeting of mRNA expression by miRNAs typically leads to faster and longer-lasting responses comparing with protein inhibition by conventional targeted therapy. Further, the ability of miRNAs to target different genes simultaneously, as it is the case for miR-320a that targets both AQP1 and AQP4, or mi29a interacting with both AQP1 and AQP3, makes another compelling point toward the development of novel AQP-targeting therapies through modulation of miRNA function. However, there are still major challenges related with miRNA application, including *in vitro* validation of *in silico* predicted miRNAs, achievement of efficient up- or down-regulation, assessment of the therapeutic effect in the most appropriate cell model and evaluation of potential off-target effects that could impair their use. Indeed, due to very small sizes, the chance that an anti-miRNA will interact with an endogenous mRNA is rather high. In addition, a hairpin RNA structure generates different miRNAs from each strand, which may bind to different mRNAs and exhibit opposite functions. Nevertheless, the possibility of using miRNAs alone or in combined therapy with other chemical or biological drugs to modulate specific AQP proteins involved in disease provides new clues for AQP-based therapeutics.

## Author contributions

GS and RC conception and design of research; IdS and AG prepared figures; AG and IdS drafted the manuscript; GS, RC, and CR edited and revised manuscript; AG, IdS, CR, RC, and GS approved final version of manuscript.

### Conflict of interest statement

The authors declare that the research was conducted in the absence of any commercial or financial relationships that could be construed as a potential conflict of interest.
